# Salvage radiotherapy for recurrent hypopharyngeal and laryngeal squamous cell carcinoma (SCC) after first-line treatment with surgery alone: a 10-year single-centre experience

**DOI:** 10.1186/s13014-019-1238-8

**Published:** 2019-02-19

**Authors:** Sati Akbaba, Thomas Held, Kristin Lang, Juliane Hoerner-Rieber, Karim Zaoui, Tobias Forster, Stefan Rieken, Peter Plinkert, Juergen Debus, Sebastian Adeberg

**Affiliations:** 1 Department of Radiation Oncology, University Hospital Heidelberg, Im Neuenheimer Feld 400, 69120 Heidelberg, Germany; 2 Heidelberg Institute for Radiation Oncology (HIRO), National Center for Radiation Research in Oncology (NCRO), Im Neuenheimer Feld 400, 69120 Heidelberg, Germany; 3 Heidelberg Ion-Beam Therapy Center (HIT), Im Neuenheimer Feld 450, 69120 Heidelberg, Germany; 4 Department of Otorhinolaryngology, Head and Neck Surgery, University Hospital Heidelberg, Im Neuenheimer Feld 400, 69120 Heidelberg, Germany

**Keywords:** Squamous cell carcinoma, Recurrent hypopharynx and larynx carcinoma, Salvage radiotherapy, Function preservation

## Abstract

**Purpose:**

Salvage surgery of recurrent hypopharyngeal and laryngeal squamous cell carcinoma (SCC) results in limited local control and survival rates. As a result of recent technological progress, radiotherapy (RT) has become a valuable, potentially curative therapeutic option. Thus, we aimed to determine prognostic factors for survival outcome in order to optimize patient selection for salvage radiotherapy after failure of first-line treatment with surgery alone in this special patient cohort.

**Methods:**

Seventy-five patients (85% male, median age of 64 years) underwent salvage RT in a secondary setting for recurrent hypopharyngeal or laryngeal SCC after prior surgery alone between 2007 and 2017. On average, patients were treated with one prior surgery (range 1–4 surgeries). Median time between surgery and salvage RT was 7 months (range 1–47 months) for initially advanced tumors (T3/4, N+, extracapsular spread) and 18 months (range 5–333 months) for initially early stage tumors. The majority of patients received concomitant chemotherapy (*n* = 48; 64%) or other kind of systemic treatment concurrent to radiotherapy (*n* = 10; 13%).

**Results:**

Median follow-up was 41 months (range 3–120 months). Overall, fifteen patients were diagnosed with local failure (all were in-field) at last follow-up (20%). Median time to recurrence was 35 months (range 3–120 months) and 3-year local progression-free survival (LPFS) was 75%, respectively. Dose-escalated RT with 70.4 Gy applied in 2.1 Gy or 2.2 Gy fractions corresponding an EQD2 > 70 Gy (*p = 0.032*) and the use of concomitant cisplatin weekly chemotherapy (*p = 0.006*) had a significant positive impact on LPFS. 3-year OS and DPFS were 76 and 85%, respectively. No toxicity-related deaths occurred. Reported grade > 3 side effects were rare (*n* = 4/70, 6%).

**Conclusion:**

Salvage radiotherapy resulted in excellent local control rates while radiation dose and the use of cisplatin weekly chemotherapy were identified as prognostic factors for LPFS. Nevertheless, patient selection for curative salvage treatment remains challenging.

## Background

Squamous cell carcinoma (SCC) of the larynx and hypopharynx is the most common tumor in the head and neck region, mainly observed in males over 50 years. Especially glottic larynx carcinomas are mostly diagnosed in early stages due to the disorder of essential laryngeal and hypopharyngeal functions presenting with initial symptoms, i.e. hoarseness, swallowing difficulties or dyspnea, resulting in significant reduced patient′s satisfaction and quality of life [1–3]. Therefore, besides achieving optimum local control, preservation of phonatory and swallowing function gain in importance. The role of chemoradiotherapy (CRT) in the organ-preserving treatment of hypopharyngeal and laryngeal malignancies was established by two important landmark trials, showing equal survival rates compared with surgery and a high rate of larynx-preservation in two-thirds of the patients [4, 5]. Over the last two decades, CRT is increasingly considered as a valuable alternative to total laryngectomy for advanced tumors. Nowadays, total laryngectomy is mostly used in highly selected patients with advanced diseases or reserved as salvage surgery in case of treatment failure after primary CRT. In the current literature, poor prognosis is reported for patients who were treated with salvage surgery after radiotherapy failure in several studies [6, 7]. However, data concerning outcome of salvage CRT after failed first-line surgical treatment are still missing. The aim of this retrospective analysis is to assess clinical outcome in patients with recurrent hypopharyngeal and laryngeal SCC after first-line treatment with surgery alone, who received second-line RT in potentially curative intention and to determine prognostic factors for survival outcome to optimize patient selection for salvage radiotherapy.

## Materials and methods

### Evaluation

Seventy-five patients with laryngeal or hypopharyngeal SCC treated for recurrence after prior surgery at the Department of Radiation Oncology of the University Hospital Heidelberg between 2007 and 2017 were identified retrospectively and patient′s records were analysed regarding local progression-free survival (LPFS), overall survival (OS) and distant progression-free survival (DPFS). Additionally, potential prognostic factors were assessed and calculated for local control (LC), OS and DPFS.

Follow-up was performed with magnetic resonance imaging (MRI) or computed tomography (CT) 6 weeks after completion of therapy, at three-month intervals during the first 2 years after treatment, every 6 months during the third year after treatment and then, once a year. Yearly CT scans of the chest and abdominal ultrasound were performed to identify distant relapse.

Acute and late toxicity were assessed according to the Common Terminology Criteria for Adverse Events version 4.03 (CTCAE v4.03) and tumor response to the current Response Evaluation Criteria in Solid Tumors (RECIST) [8, 9]. TNM (tumor, nodal, metastasis) stage was assessed and adjusted to the eight edition of the TNM classification system [10].

Survival data were calculated from first diagnosis to the date of last follow-up, death or progression by using Kaplan-Meier estimates (IBM SPSS Statistics version 24). OS was calculated from the first diagnosis up to the last follow-up or death. LPFS and DPFS were considered as the time period between first diagnosis and occurrence of local progression, distant progression or death. LC was calculated from first day of treatment to last follow-up or local progression. Univariate analysis to identify potential prognostic factors for survival outcome were performed using the log-rank test. For multivariate analysis, the cox regression model was used. All tests were 2-tailed and the significance level was defined as *∝ < 0.05*.

### Patient characteristics

All patients received RT in a secondary setting for recurrence after first-line treatment with surgery alone (median number of operations 1, range 1–4). Patients who received a prior radiation treatment were excluded from the study. Initially, postoperative RT (*n* = 13, 17%) for UICC stage III/IV or CRT (*n* = 22, 29%) for incomplete resection margin (*n* = 11, 15%), lymph node metastases with extracapsular spread (ECS; *n* = 9, 12%) or both (n = 2, 3%) was obligatory in overall 47% of the patients but was declined from the patient. Therefore, two treatment groups were differed within the study population; early stage patients without an initial indication for postoperative RT (*n* = 40, 53%) and patients with advanced tumors who declined postoperative RT after first-line surgery (*n* = 35, 47%). Prior total laryngectomy was performed in 24% of the patients (*n* = 18), prior unilateral neck dissection (ND) in 9% of the patients (*n* = 7) and bilateral ND in 35% of the patients (*n* = 26). In 79%, lymph node metastases could be identified (n = 26), in 11/26 patients with ECS (for detailed treatment characteristics please see Table 2).

The majority of the patients were male (*n* = 64, 85%) and older than 60 years (*n* = 45, 60%) at RT initiation (median age of 64 years, range 46–83 years). The most common initial tumor sites were the glottic larynx with 61% (*n* = 46), the hypopharynx with 19% (*n* = 14) and the supraglottic larynx with 16% (*n* = 12). Regarding the recurrent tumor sites after prior surgery, the glottic larynx (*n* = 31, 41%), the neopharynx (n = 12, 16%) and the hypopharynx (n = 14, 19%) dominated. At first diagnosis, 56% of the tumors were at UICC stage I-II (*n* = 44). At recurrence, higher UICC stages could be identified with 28% for UICC stage III (*n* = 21), 25% for UICC stage IVA (*n* = 19), 13% for UICC stage IVB (*n* = 10) and 1% for UICC stage IVC (n = 1). Patient characteristics are shown in Table 1.

**Table 1:** Patient and tumor characteristics at first-line and second-line treatment, *n* = 75

characteristic	No. (%)	
	first-line treatment (surgery)	second-line treatment (RT/CRT)
median age (years)	59 (42–83)	62 (46–83)
gender		
male	64 (85)	64 (85)
female	11 (15)	11 (15)
Karnofsky performance score in %		
100	25 (33)	12 (16)
90	18 (24)	23 (31)
80	24 (32)	17 (23)
70	8 (11)	17 (23)
60	none	6 (8)
tumor site		
glottic larynx	46 (61)	31 (41)
subglottic larynx	3 (4)	10 (13)
supraglottic larynx	12 (16)	8 (11)
hypopharynx	14 (19)	14 (19)
neopharynx	none	12 (16)
UICC stage		
I	20 (27)	16 (21)
II	22 (29)	8 (11)
III	9 (12)	21 (28)
IVA	22 (29)	19 (25)
IVB	1 (1)	10 (13)
IVC	1 (1)	1 (1)
TNM stage		
T1	22 (29)	22 (29)
T2	25 (33)	12 (16)
T3	14 (19)	15 (20)
T4	14 (19)	26 (35)
N0	51 (68)	48 (64)
N1	3 (4)	11 (15)
N2	20 (27)	14 (19)
N3	1 (1)	1 (1)
M0	74 (99)	73 (97)
M1	1 (1)	2 (3)
G stage		
G1	2 (3)	2 (3)
G2	51 (68)	49 (65)
G3	19 (25)	21 (28)
Gx	3 (4)	3 (4)

*abbreviations*: *RT* radiotherapy, *CRT* chemoradiotherapy, *T* tumor stage, *N* nodal stage, *M* metastasis stage, *G* grading, *UICC* Union Internationale Contre le Cancer

### Treatment characteristics

For treatment planning, CT and MRI were performed and patients were immobilized with custom-made thermoplastic masks with shoulder fixation. Clinical target volume 1 (CTV1) including the macroscopic tumor and the potential microscopic spread and CTV2 including the CTV1 and the lymphatic drainage were outlined. Planning target volumes (PTVs) were generated with a margin of 5 mm around the CTVs and received at least 90% of the prescription dose. All patients received intensity modulated radiotherapy (IMRT) via tomotherapy with (*n* = 70, 93%) or without simultaneous integrated boost (SIB, *n* = 5, 7%) at a median time of 7 months after the first operation (range 1–333 months). Single doses and prescription doses differed, thus we calculated the equivalent dose in 2 Gy per fraction with the following formula for better dose comparibility: EQD2 = D x ((d + α/β)/(2 + α/β)) (D = total dose in Gy, d = single dose in Gy, α/β = 2).

The median prescribed total dose was 54 Gy (range 52.2–66 Gy) with a median single dose fraction of 1.8 Gy (range: 1.8–2.0 Gy) to the bilateral cervical lymph drainage and 66 Gy (range 58–72 Gy) with a median single dose fraction of 2.2 Gy (range 1.8–2.2 Gy) to the macroscopic tumor. The median equivalent dose to a 2 Gy single dose fraction (EQD2) prescribed on the macroscopic tumor was 67 Gy (range 60–73 Gy). In 5 cases, treatment could not be finished (7%). Thus, these patients received smaller cumulative doses. The median PTV1 was 101 cc (range 21–222 cc) and the median PTV2 was 740 cc (range 51–1301 cc). Overall, 77% of the patients were treated with concomitant systemic therapy (*n* = 58) of whom 64% received concomitant chemotherapy (*n* = 48) with carboplatin/5-fluoruracil in week 1 and 5 (n = 4) or cisplatin weekly (*n* = 44) and 13% concomitant immunotherapy with cetuximab weekly (*n* = 10). Treatment characteristics are depicted in Table 2.

**Table 2:** Treatment characteristics at first-line and second-line treatment, n = 75

first-line treatment (surgery)	
number of previous surgeries	
1	46 (61)
2	17 (23)
3	10 (13)
4	2 (3)
total laryngetomy	18 (24)
larynx-preserving surgery	57 (76)
vocal cord tripping	2 (3)
endoscopic laser resection	21 (28)
partial laryngectomy	34 (45)
unilateral ND	10 (13)
bilateral ND	27 (36)
ECS	11 (30)
second-line treatment (RT/CRT)	
median EQD2 in Gy	67 Gy (60–72 Gy)
median PTV1	101 cc (21–1949 cc)
median PTV2	740 cc (51–72 cc)
concomitant chemotherapy	48 (64)
concomitant cetuximab	10 (13)

*abbreviations*: *RT* radiotherapy, *CRT* chemoradiotherapy, *ECS* extracapsular spread, *EQD2* equivalent dose in 2 Gy single dose fractions, *ND* neck dissection, *PTV* planning target volume

## Results

### Survival analysis

Five patients could not finish therapy for deterioration of their general condition (KPS < 60%) and were lost to follow-up after treatment. Therefore, we excluded these patients from the analysis (7%). Median follow-up for all remaining patients was 41 months (range 8–120 months) and for surviving patients 50 months (range 8–120 months). At last follow-up, 56% of the patients were still alive (*n* = 39) of whom 87% were free of local failure (*n* = 34). Median OS from first diagnosis up to last follow-up or death was 59 months (range 11–373 months) and from RT up to last follow-up or death 43 months (range 14–121 months). Overall, complete remission was seen in 48 patients (64%) and partial remission in six patients (8%). 20% of the patients developed an in-field recurrence (*n* = 14) and one patient a locoregional recurrence in cervical lymph nodes (1%) after a median time of 12 months (range 3–19 months) after RT. All local and locoregional recurrences occurred within the first 2 years after RT. Distant metastases (*n* = 13) were diagnosed in 19% of the patients after a median time of 28 months (range 2–88 months) after the first diagnosis with pulmonary failure in 11 cases (16%) and bone metastases in 2 cases (3%). Corresponding 3-year and estimated 5-year LPFS, OS and DPFS were 75, 76, 85 and 75%, 64, 82% for all patients without significant differences for initially early stage (without an initial indication for adjuvant RT) vs. advanced stage (with an initial indication for adjuvant RT) larynx/hypopharynx tumors in the LPFS (*p = 0.431*; HR = 1.12; 95%-CI = 0.38–21.30), OS (*p = 0.518*; HR = 0.89; 95%-CI = 1.24–12.46) and DPFS (*p = 0.081*; HR = 3.04; 95%-CI = 1.98–8.34), respectively. Patients with local or locoregional recurrence received either palliative systemic therapy (*n* = 5, 7%), best supportive care (*n* = 1, 1%), salvage larynx-preserving surgery (*n* = 2, 3%), salvage ND (n = 1, 1%), salvage total laryngectomy (n = 5, 7%) or re-RT (n = 1, 1%). Thus, preservation of the larynx could be achieved in 90% (*n* = 47/52) of the patients apart from patients who were initially treated by total laryngectomy.

### Prognostic factors for survival

We analysed the impact of potential prognostic factors at time of first-line treatment with surgery alone (initial T, N, G stage, ECS, tumor site, Karnofsky performance status (KPS), prior laryngectomy vs. larynx-preserving surgery, prior ND yes vs. no, time period between first operation and first recurrence) and at time of second-line treatment with salvage RT (recurrent T, N, G stage, KPS, number of prior operations, EQD2 > 70 Gy vs. ≤70 Gy, PTV1 ≥ 101 cc vs. < 101 cc, cisplatin weekly chemotherapy vs. others, local recurrences yes vs. no after RT and metastases yes vs. no) on OS, LPFS and DPFS. Several potential prognostic factors were identified using the log-rank test for univariate analyses. Independent prognostic factors were assessed by the cox regression model for multivariate analysis. The results of univariate and multivariate analysis are shown in Table 3.

**Table 3:** Results (*n* = 70)

characteristics	5-year survival	univariate analysis		multivariate analysis	
		HR (95%-KI)	*p* value	HR (95%-KI)	*p* value
overall survival					
at first-line treatment (surgery)					
iT stage (T3/4 vs. T1/2)	45% vs. 72%	1.46 (1.01–2.11)	0.041		
iN stage (N+ vs. N0)	25% vs. 78%	5.78 (2.66–12.59)	0.000	2.50 (0.96–6.49)	0.060
at second-line treatment (RT/CRT)					
recurrent tumor site (others vs.					
glottis)	42% vs. 89%	4.70 (1.79–12.33)	0.001	4.77 (1.00–22.70)	0.050
rKPS (≥90% vs. < 90%)	68% vs. 36%	0.55 (0.35–0.84)	0.050		
chemotherapy (others vs. cisplatin weekly)	35% vs. 64%	2.37 (1.04–5.45)	0.035		
local recurrence after RT					
(yes vs. no)	25% vs. 74%	3.10 (1.44–6.67)	0.002	*2.63 (1.04–6.69)*	*0.041*
metastases after RT (yes vs. no)	3% vs. 70%	3.44 (1.54–7.71)	0.001		
local progression-free survival					
at first-line treatment (surgery)					
iN stage (N+ vs. N0)	50% vs. 86%	4.52 (1.60–12.72)	0.002	3.62 (0.92–14.19)	0.065
iG stage (G3 vs. G1/2)	59% vs. 82%	2.77 (1.00–7.66)	0.038		
at second-line treatment (RT/CRT)					
rG stage (G3 vs. G1/2)	59% vs. 82%	2.66 (1.04–7.80)	0.036		
EQD2 (> 70 Gy vs. =70 Gy)	90% vs. 68%	0.25 (0.06–1.10)	0.045	*0.10 (0.01–0.82)*	*0.032*
chemotherapy (others vs. cisplatin weekly)	46% vs. 86%	5.78 (1.75–19.13)	0.001	*3.62 (0.92–14.19)*	*0.006*
distant progression-free survival					
at first-line treatment (surgery)					
iN stage (N+ vs. N0)	40% vs. 89%	6.19 (1.85–20.76)	0.001	*2.56 (1.03–6.34)*	*0.042*
iG stage (G3 vs. G1/2)	65% vs. 85%	3.06 (1.03–9.13)	0.035	3.45 (0.98–12.23)	0.055
at second-line treatment (RT/CRT)					
rT stage (T3/4 vs. T1/2)	70% vs. 90%	3.30 (0.91–11.99)	0.055	*6.02 (1.29–30.00)*	*0.028*
rG stage (G3 vs. G1/2)	65% vs. 85%	3.12 (1.00–9.25)	0.037	3.45 (0.98–12.23)	0.054
recurrent tumor site (others vs.					
glottis)	68% vs. 100%	6.02 (0.68–10.24)	0.014		
PTV1 volume (=median vs.					
<median of 101 cc)	65% vs. 87%	3.85 (1.04–14.22)	0.030	*5.39 (1.65–12.53)*	*0.038*
chemotherapy (others vs. cisplatin weekly)	57% vs. 82%	3.27 (1.03–10.41)	0.034		

*abbreviations*: *EQD2* equivalent dose to 2 Gy single fraction, *HR* hazard ratio, *iT stage* initial tumor stage, *iN stage* initial nodal stage, *KPS* Karnofsky performance score, *RT* radiotherapy, *CRT* chemoradiotherapy, *G stage* grading stage, *rT stage* recurrent tumor stage, *PTV* planning target volume

**Fig. 1:**
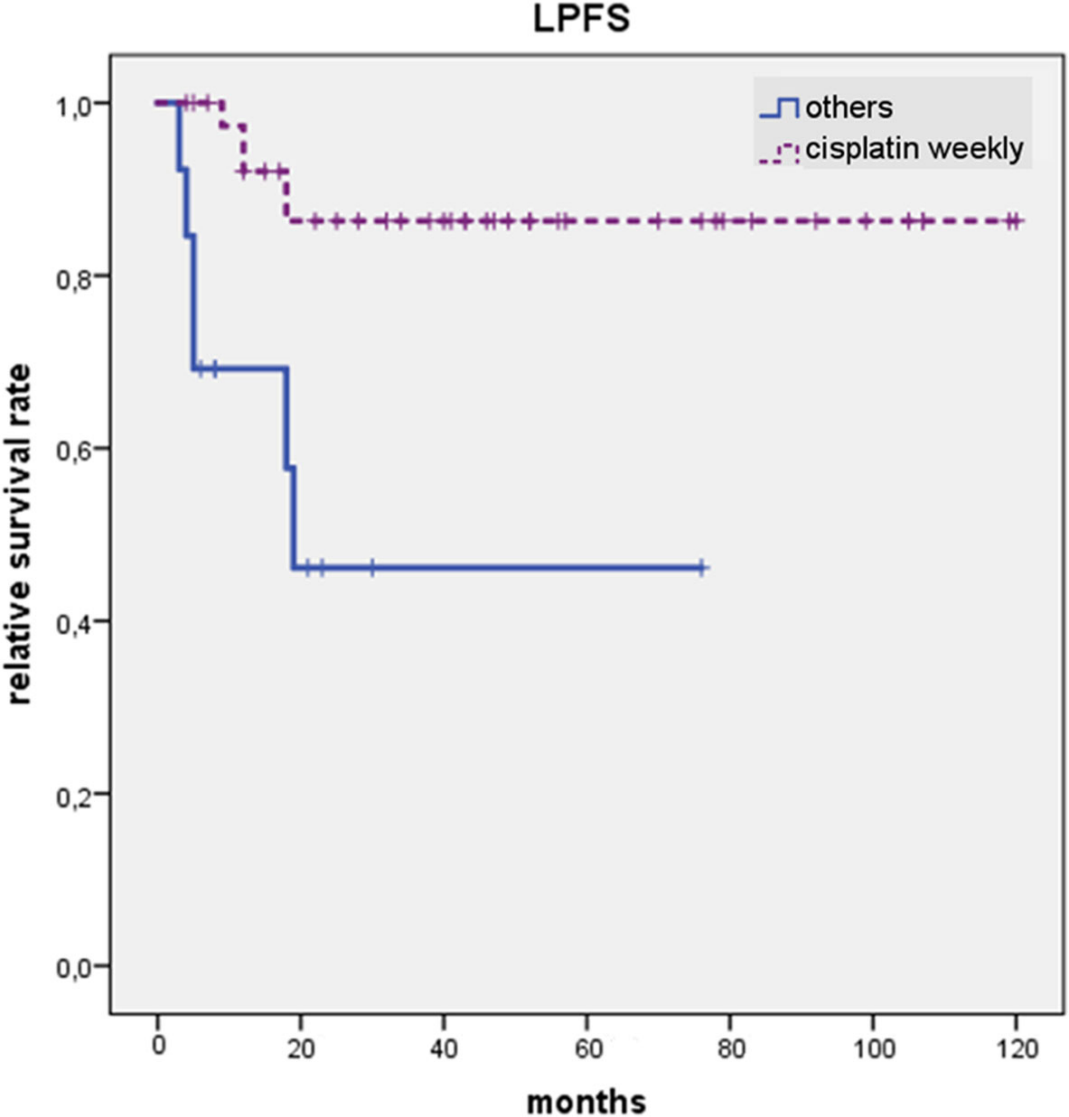
LPFS depends significantly on the use of concomitant systemic therapy with survival benefit for patients receiving cisplatin weekly chemotherapy vs. others (carboplatin/5-fluoruracil and cetuximab) (*p* = 0.006). 5-year LPFS amounts 86% vs. 43% for patients who received concomitant cisplatin weekly vs. patients who did not

**Fig. 2:**
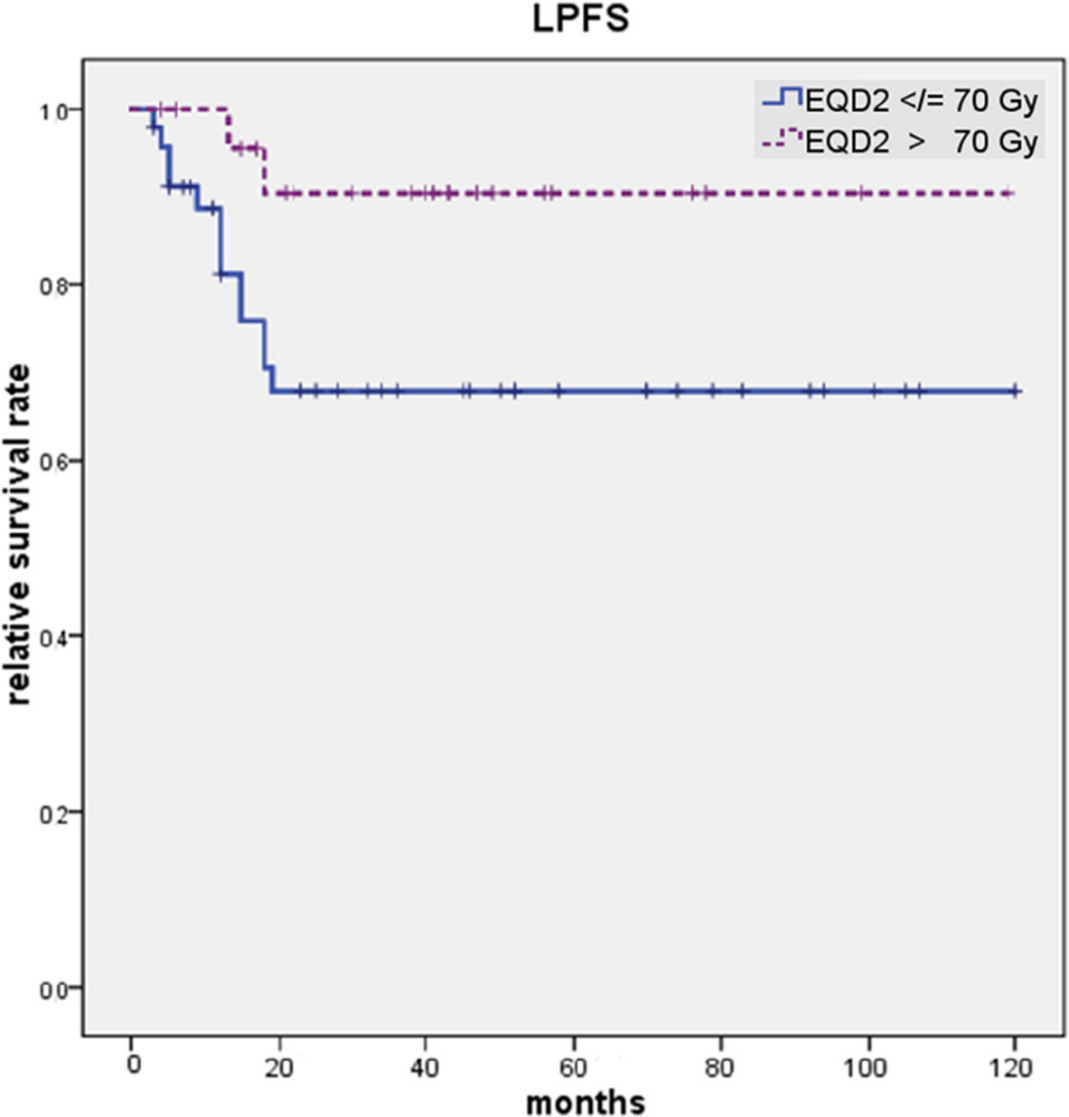
LPFS depends significantly on the applied RT dose with survival benefit for patients receiving an EQD2 > 70 Gy (*p* = 0.032). Patients who received an EQD2 > 70 Gy show a 5-year LPFS of 90% vs. 68% compared with patients who received an EQD2 ≤ 70 Gy

Regarding LPFS, we could identify the use of concomitant chemotherapy with cisplatin weekly (*p = 0.006*) and an EQD2 > 70Gy prescribed on the macroscopic tumor as positive prognostic factors (*p = 0.032*). Patients who received concomitant systemic therapy were included into analysis only. The use of concomitant chemotherapy with cisplatin weekly resulted in a 5-year LPFS of 86% vs. 44% compared with patients who received concomitant chemotherapy with carboplatin/5-FU in week 1 and 5 or cetuximab weekly (Fig. 1). With regard to the applied EQD2 on the macroscopic recurrent tumor, patients who received an EQD2 > 70 Gy had a 5-year LPFS of 90% vs. 68% compared with patients who received an EQD2 ≤ 70 Gy (Fig. 2).

Patients with local or locoregional recurrence after salvage RT had a significant worse OS with a 5-year OS of 25% vs. 74% estimated from Kaplan-Meier analysis compared with patients who were free from recurrence at last follow-up (*p = 0.041*), respectively. Furthermore, recurrent tumor site showed an estimated impact on patients′ OS. Thus, patients with glottic recurrence had a survival benefit with a 5-year OS rate of 89% compared with patients who had recurrences in the area of the supraglottic or subglottic larynx, hypopharynx or neopharynx with a 5-year OS rate of 25% *(p = 0.05)*. Additionally, initial N stage seemed to have a further impact on OS with a survival benefit for patients without initial lymph node metastases, but in the multivariate analysis, the significance level could not be achieved (*p = 0.06*).

In terms of DPFS, recurrent T stage showed the most significant impact on DPFS with a 5-year DPFS of 90% for rT1/2 tumors and 70% for rT3/4 tumors (*p = 0.028*). Further, PTV1 (≥ median of 101 cc vs. < median of 101 cc) had a worse impact on DPFS with increasing volume (*p = 0.038*). At least, initial N stage could be diagnosed as further prognostic factor for DPFS with a significant lower occurrence of metastases for iN0 stage compared with iN+ stage (*p = 0.042*). Increasing N+ stage (N1 vs. N2 vs. N3) had no impact on DPFS (*p = 0.140*).

### Toxicity

Overall, 20% of the patients reported acute grade 3 (*n* = 14) and 19% of the patients chronic grade 3 and grade 4 toxicity (*n* = 13). During and six weeks after therapy, no acute grade 4 toxicity could be identified. An overview of acute and late side effects is shown in Table 4. Acute grade 3 toxicity consisted of mucositis (*n* = 2, 3%), dysphagia (6%, *n* = 4), odynophagia (*n* = 5, 7%), dermatitis (n = 4, 6%), xerostomia (n = 1, 1%), hoarseness (*n* = 3, 4%) and lymphedema (n = 1, 1%). Three months after therapy, the majority of the acute grade 3 side effects disappeared. Nevertheless, 3 patients received tracheostomy for acute dyspnea due to grade 4 lymphedema of the larynx (4%) and in one patient a laryngoesophageal fistula could be diagnosed 6 months after therapy (1%). 3 patients claimed about chronic severe dysphagia with high-grade stenosis of the laryngoesophageal junction with the need of regular bougienage up to the last follow-up (4%). Further, one patient developed a laryngocutaneous fistula 12 months after RT (1%) and one patient developed a wound healing disorder in radiation field 24 months after therapy (1%).

**Table 4:** Overview of acute and chronic toxicity (n = 70)

characteristic	acute toxicity (n = 70)	chronic toxicity (*n* = 63)			
	No. (%)	No. (%)			
	under RT and 6 weeks post RT	3–6 months post RT	12 months post RT	24 months post RT	at last follow-up
toxicity					
grade 1	10 (14)	19 (27)	22 (31)	23 (33)	17 (24)
grade 2	39 (56)	21 (30)	10 (14)	6 (9)	5 (7)
grade 3	14 (20)	4 (6)	3 (4)	3 (4)	3 (4)
grade 4	0	4 (6)	1 (1)	1 (1)	
dysphagia					
grade 1	6 (9)	7 (10)	6 (9)	6 (9)	5 (7)
grade 2	28 (40)	4 (6)	5 (7)	1 (1)	0
grade 3	4 (6)	3 (4)	3 (4)	3 (4)	3 (4)
odynophagia					
grade 1	8 (11)	1 (1)	0	0	0
grade 2	14 (20)	0	0	0	0
grade 3	5 (7)	0	0	0	0
mucositits					
grade 1	12 (17)	1 (1)	0	0	0
grade 2	36 (51)	0	0	0	0
grade 3	2 (3)	0	0	0	0
dermatitis					
grade 1	19 (27)	2 (3)	0	0	0
grade 2	18 (26)	1 (1)	0	0	0
grade 3	4 (6)	0	0	0	0
xerostomia					
grade 1	32 (46)	30 (43)	17 (24)	13 (19)	10 (14)
grade 2	12 17 ()	4 (6)	2 (3)	2 (3)	2 (3)
grade 3	1 (1)	0	0	0	0
hoarseness					
grade 1	16 (23)	15 (21)	13 (19)	12 (17)	10 (14)
grade 2	13 (19)	7 (10)	3 (4)	1 (1)	2 (3)
grade 3	3 (4)	1 (1)	1 (1)	1 (1)	0
fatigue					
grade 1	6 (9)	3 (4)	2 (3)	1 (1)	1 (1)
grade 2	6 (9)	2 (3)	1 (1)	0	0
dysgeusia					
grade 1	11 (16)	14 (20)	11 (16)	8 (11)	7 (10)
grade 2	20 (29)	4 (6)	0	0	0
dry cough					
grade 1	7 (10)	5 (7)	2 (3)	2 (3)	0
grade 2	1 (1)	0	0	0	0
lymphedema					
grade 1	6 (9)	12 (17)	2 (3)	1 (1)	1 (1)
grade 2	8 (11)	2 (3)	1 (1)	1 (1)	4 (6)
grade 3	1 (1)	1 (1)	1 (1)	1 (1)	1 (1)
grade 4^a^	0	3 (4)	0	0	0
fistula					
laryngoesophageal	0	1 (1)	0	0	0
laryngocutaneous	0	0	1 (1)	0	0
wound healing disorder	0	0	0	1 (1)	0
gastric tube dependence	24 (34)	9 (13)	5 (7)	1 (1)	1 (1)

*abbreviations*: *RT* radiotherapy

^a^with tracheotomy for acute dyspnea

Under RT, 43% of the patients needed supportive nutrition for nutritional difficulties due to acute side effects (*n* = 30). Acute gastric tube dependence counted 34% (*n* = 24). 3 to 6 months after therapy, only 13% of the patients needed farther a gastric tube for nutrition (*n* = 9). Only one patient was dependent on a gastric tube for more than 2 years (1%).

## Discussion

### Findings

The majority of the patients in the current analysis were treated with dose-escalated RT (93%) and concomitant cisplatin weekly chemotherapy (60%) for recurrent laryngeal or hypopharyngeal SCC after first-line treatment with surgery alone. Two thirds of the patients had tumors in advanced stages (UICC III and IV) before treatment. Nevertheless, we identified an excellent 3-year LPFS, OS and DPFS of 75, 76 and 85% and an estimated 5-year LPFS, OS and DPFS of 75, 64 and 82%, respectively. The 5-year local control rate was 79% for this unfavourable patient population. All recurrences occurred in-field and within the first two years after treatment. Larynx-preservation could be observed in 90% of the patients who received organ-preserving surgery as initial treatment. Local control was best achieved in patients receiving an EQD2 > 70 Gy on the macroscopic tumor and concomitant chemotherapy with cisplatin weekly. RT dose and the use of cisplatin chemotherapy did not correlate with patients’ KPS (*p = 0.139*). OS depended negatively on the occurrence of local recurrences after salvage RT and on the recurrent tumor site before RT with survival benefit for recurrent tumors involving the glottis. DPFS differed significantly regarding recurrent tumor size and the initial nodal stage. Overall, compliance was well (93%) and RT was tolerated with moderate toxicity.

### Survival results

Nowadays, CRT is seen as an equivalent therapy option to surgery regarding tumor control in laryngeal and hypoharyngeal tumors [11, 12]. Thus, Mendenhall et al. reported 2001 RT alone for T1/2 N0 larynx tumors as an alternative curative therapy option to surgery with a comparable 5-year LPFS of 72 to 94% depending on T stage, a 5-year OS of 79% and a 5-year DPFS of 98% [13]. Nevertheless, surgery in form of organ-preserving endoscopic resection, laser surgery or open-neck partial laryngectomy is still considered as the gold standard in the treatment of early stage tumors with local control rates of 60 to 95%, declining with increasing T stage [12]. While local relapses are relatively rare after primary treatment of early stage tumors, local control in advanced hypopharyngeal and laryngeal tumors remains a challenge. As organ-preserving treatment strategies are increasingly used as first-line treatment, the use of organ-preserving RT in combination with chemotherapy for advanced stages gained in importance within the last decades [5, 13, 14]. However, 40 to 60% of patients with advanced tumors relapse after primary CRT [5, 15]. Salvage treatment options for these patients are limited, as re-irradiation is mostly limited by the tumor site and the necessary prescription dose to the recurrent tumor. In these cases, salvage surgery is mostly required. The effectiveness of salvage surgery after failure of primary CRT (2-year OS between 27 and 71%) is reported by several studies [16–18]. Taguchi et al. reported a 5-year OS and disease-specific survival of 61 and 66% for salvage surgery after primary CRT vs. 10 and 10% for patients who failed primary CRT but did not undergo salvage surgery [18]. While poor prognosis with a 5-year OS of 16% is described for recurrent hypopharyngeal tumors after salvage surgery, the same treatment method offers 5-year OS rates ranging from 57 to 70% for recurrent laryngeal tumors [19–21]. Recurrent hypopharyngeal tumors are mostly considered inoperable and should, therefore, be treated with alternative salvage methods [22]. Salvage CRT is generally reserved for recurrent tumors after primary total laryngectomy, for patients with inoperable recurrent tumors or in cases, where the patient rejects total laryngectomy as the only remaining treatment option in order to further preserve the organ function. Patients who receive salvage CRT mostly appear with initially early stage tumors and represent a non-comparable patient collective to patients who receive salvage surgery after failure of primary CRT. Nevertheless, studies describing a homogeneous patient population after salvage CRT including recurrent hypopharyngeal and laryngeal tumors only are lacking. Lee et al. reported a 2-year OS and progression-free survival of 74 and 68% for patients with recurrent hypopharyngeal and laryngeal tumors who underwent different salvage treatment methods, i.e. salvage surgery with or without RT, RT alone, chemotherapy alone, after different initial treatments, i.e. CRT for advanced tumors or primary surgery for early stage tumors [23]. Li et al. analysed patients who were initially treated with surgery, RT or CRT for recurrent laryngeal tumors. The patients received salvage treatment with surgery for operable recurrent tumors in 54% and radiation in 16% of the cases [24]. Salvage surgery resulted in a 5-year OS rate of 73% vs. 32% for other salvage treatment methods like RT, while the high OS rate for salvage surgery and the decreased OS of salvage RT were discussed via patient selection bias (operable tumors, initial tumor stage). This could be a valuable reason for the excellent survival results in the current analysis as well, as the majority of our patients had initially early stage tumors (56% UICC I/II). Nevertheless, survival analysis showed no significant difference in the LPFS, OS and DPFS for initially early stage and initially advanced tumors.

### Prognostic factors

Several meta-analyses and randomized studies have proven the beneficial role of concomitant chemotherapy in combination with RT, especially regarding cisplatin chemotherapy, showing superior local control and OS rates in laryngeal/hypopharyngeal tumors as well as in other HNC either in the primary or postoperative setting [5, 15, 25–27]. Besides an EQD2 > 70 Gy achieved by using fractionation doses >2Gy, we could identify a significant impact of cisplatin chemotherapy on LPFS only [28, 29]. Nevertheless, Pignon et al. could show in the MACH-NC meta-analysis that chemotherapy with carboplatin and 5-fluoruracil is considered to be equivalent to cisplatin chemotherapy. The results of the current study could possibly be explained by patient selection bias between both groups [30]. In the current literature, further factors influencing LPFS, i.e. T stage, N stage, G stage, sex, age, vocal cord invasion, overall treatment time and RT field size are discussed in the first-line treatment [13, 31–34]. Glottic tumors seem to have a survival benefit compared with other tumor sites, thus in several studies superior OS rates are described [17–20]. The occurrence of local recurrence after treatment, tumor size and N stage are accepted as further prognostic factors [31–35]. For second-line treatment, initial tumor in the hypopharynx vs. the larynx, recurrent tumor in the hypopharynx vs. the larynx, advanced primary tumor, advanced recurrent tumor, advanced primary N stage and advanced initial and recurrent G stage were most frequently associated with decreased progression-free survival resulting in a poor prognosis [23, 24, 36]. In multivariate analysis, we could not identify an impact of tumor size and N stage on OS, possibly due to the low patient number but on DPFS. Thus, tumors with initial N+ stage and increased recurrent tumor size (PTV1, rT stage) influenced distant control negatively [34]. Additionally, tumor differentiation (G stage) can be considered as further prognostic factor regarding DPFS [31].

### Toxicity

Superior dose conformity and decreased toxicity due to improved preservation of organs at risk compared with 3D-RT is described for IMRT [37–40]. Nevertheless, toxicity after IMRT remains high [41–45]. Especially in the hypopharynx and larynx region, significant higher rates of late side effects occur due to the proximity of several organs at risk compared with other regions of the head and neck [45]. For patients treated with concurrent CRT, Forastiere et al. could show in the RTOG trial 91–11 a high rate of severe late grade 3 and 4 toxicity, especially regarding mucositis (43%) [44]. In a RTOG analysis of three RTOG trials (RTOG 91–11, RTOG 97–03, RTOG 99–14), Machtay et al. identified 43% late side effects after CRT for locally advanced squamous cell carcinoma of the head and neck, and described grade 3 and 4 pharyngeal and laryngeal dysfunction in 39%, gastric tube dependence longer than 2 years in 13% and treatment-related death within 3 years in 10% of the patients. Swallowing limitations, aspiration, laryngoesophageal stricture and dysphagia dominated regarding laryngoesophageal dysfunction [45]. Caudell et al. reported a 3-year laryngoesophageal dysfunction-free survival (LEDFS) of 32% for patients who were treated with CRT for advanced SCC of the larynx and hypopharynx [42]. For salvage treatment methods as well, high toxicity rates are reported. Several authors described complication rates between 44 and 59% for salvage surgery after first-line CRT [16, 17, 46]. A systematic review and meta-analysis of the complications of salvage total laryngectomy including 3293 patients by Hasan et al. showed a complication rate of 68% with 29% pharyngocutaneous fistula [47].In the current analysis, in contrast, only 19% of the patients claimed about severe chronic toxicity. Especially laryngoesophageal dysfunction consisting of severe dysphagia (4%), fistula (3%), dyspnea (4%) or wound healing disorder (1%) were observed. Only one patient showed feeding tube dependence over 2 years after RT (1%). We could not identify any treatment-related deaths within the follow-up time. Despite first-line surgery and second-line dose-escalated RT, we identified moderate toxicity rates making salvage CRT after failed surgery a safe therapy alternative.

## Conclusion

Salvage radiotherapy is an effective curative therapy option for recurrent hypopharyngeal and laryngeal SCC after prior surgery with excellent local control rates and moderate toxicity comparable to prior results concerning primary radiotherapy. We recommend dose-escalated IMRT with an EQD2 > 70 Gy as well as the use of concomitant cisplatin weekly chemotherapy for superior LPFS. Nevertheless, patient selection for curative salvage treatment remains challenging.
